# Evidence of Increased Muscle Atrophy and Impaired Quality of Life Parameters in Patients with Uremic Restless Legs Syndrome

**DOI:** 10.1371/journal.pone.0025180

**Published:** 2011-10-03

**Authors:** Christoforos D. Giannaki, Giorgos K. Sakkas, Christina Karatzaferi, Georgios M. Hadjigeorgiou, Eleftherios Lavdas, Vassilios Liakopoulos, Nikolaos Tsianas, Georgios N. Koukoulis, Yiannis Koutedakis, Ioannis Stefanidis

**Affiliations:** 1 Department of Nephrology, University of Thessaly, Larissa, Thessaly, Greece; 2 Department of Neurology, University of Thessaly, Larissa, Thessaly, Greece; 3 Department of Endocrinology, School of Medicine, University of Thessaly, Larissa, Thessaly, Greece; 4 Department of Medical Radiological Technologists, Technological Educational Institute of Athens, Athens, Attika, Greece; 5 Department of Sport Science, University of Thessaly, Trikala, Thessaly, Greece; 6 Centre for Research and Technology –Thessaly, Volos, Thessaly, Greece; 7 Department of Nephrology, General Hospital of Trikala, Trikala, Thessaly Greece; 8 Department of Life and Health Sciences, University of Nicosia, Nicosia, Cyprus; Mayo Clinic, United States of America

## Abstract

**Background:**

Restless Legs Syndrome is a very common disorder in hemodialysis patients. Restless Legs Syndrome negatively affects quality of life; however it is not clear whether this is due to mental or physical parameters and whether an association exists between the syndrome and parameters affecting survival.

**Methodοlogy/Principal Findings:**

Using the Restless Legs Syndrome criteria and the presence of Periodic Limb Movements in Sleep (PLMS/h >15), 70 clinically stable hemodialysis patients were assessed and divided into the RLS (n = 30) and non-RLS (n = 40) groups. Physical performance was evaluated by a battery of tests: body composition by dual energy X ray absorptiometry, muscle size and composition by computer tomography, while depression symptoms, perception of sleep quality and quality of life were assessed through validated questionnaires. In this cross sectional analysis, the RLS group showed evidence of thigh muscle atrophy compared to the non-RLS group. Sleep quality and depression score were found to be significantly impaired in the RLS group. The mental component of the quality of life questionnaire appeared significantly diminished in the RLS group, reducing thus the overall quality of life score. In contrast, there were no significant differences between groups in any of the physical performance tests, body and muscle composition.

**Conclusions:**

The low level of quality of life reported by the HD patients with Restless Legs Syndrome seems to be due mainly to mental health and sleep related aspects. Increased evidence of muscle atrophy is also observed in the RLS group and possibly can be attributed to the lack of restorative sleep.

## Introduction

Restless Legs Syndrome (RLS) is a sensory-motor neurological disorder characterized by an irresistible urge to move one's extremities. This unpleasant sensation becomes worse during inactivity and especially at night [1]. RLS is a very common feature among patients receiving hemodialysis (HD) therapy [2]–[4] and in this case it is called uremic RLS. In uremic patients, RLS has been associated with poorer quality of life (QoL) compared to RLS free patients [5]–[8], however it is not clear whether the impaired QoL is due to physical or mental aspects of the QoL.

Reductions in physical performance and physical functioning, and increasing restraints to independent living are affecting QoL in HD population [9], whereas muscle wasting and low lean body mass are additionally associated to lower survival rates [10], [11]. It has been reported that HD-RLS patients have a higher mortality rate compared to their non RLS counterparts [8], [12]. However, it is still unknown whether HD patients with RLS experience any further declines in parameters directly affecting survival such as body composition, muscle quality and quantity, physical performance and functional capacity, compared to RLS free patients.

The possible effect of RLS on these parameters is ambiguous. On one hand, the discomfort that the syndrome induces to the patients, may lead to avoidance and lack of exercise. On the other hand, the repeated movement-relaxation cycle that the RLS patients experience in order to get relief from the symptoms could lead to a relatively increased physical activity. Nevertheless, both scenarios could affect body composition as well as the quality and quantity of muscle tissue, influencing thus the patients' mortality and morbidity rates.

Moreover, RLS, either in its idiopathic [13] or uremic [6] form, is well known to impair patients' sleep. Disturbed sleep is reported to be associated with reductions in circulating anabolic hormones [14], [15] thus it could affect muscle metabolism. Indeed, some data indicates that the lack of sleep is associated with reduced muscle size in HD patients [16] and it is plausible that in HD patients with RLS the anabolic effect of sleep might be further reduced. Given that RLS disturbs sleep quality, we hypothesized that this could have a detrimental effect in the HD patient's muscle quantity and/or quality.

Given the high prevalence of undiagnosed RLS in HD patients,[5], [17] and the fact that RLS by nature could influence aspects of physical performance, physical functioning and QoL, such as endurance, body composition and muscle size, it is conceivable to assume that research outcomes based on HD populations could have been confounded in the past.

The aims of the present study were to investigate whether HD patients with RLS, as compared to those without the syndrome, show evidence of further reduced quality of life and of overall physical performance and functional capacity, as well as further alterations in indices of body composition and muscle characteristics, parameters that could have a significant impact in the survival and quality of life in this patient- population.

## Methods

### Ethics Statement

The study was approved by the Ethics Committee of the University of Thessaly, and by the bioethics committee of the University General Hospital of Larissa, Greece and the bioethics committee of the General Hospital of Trikala, Greece. All patients gave their written informed consent prior to study participation.

### Study Population

**Figure 1:**
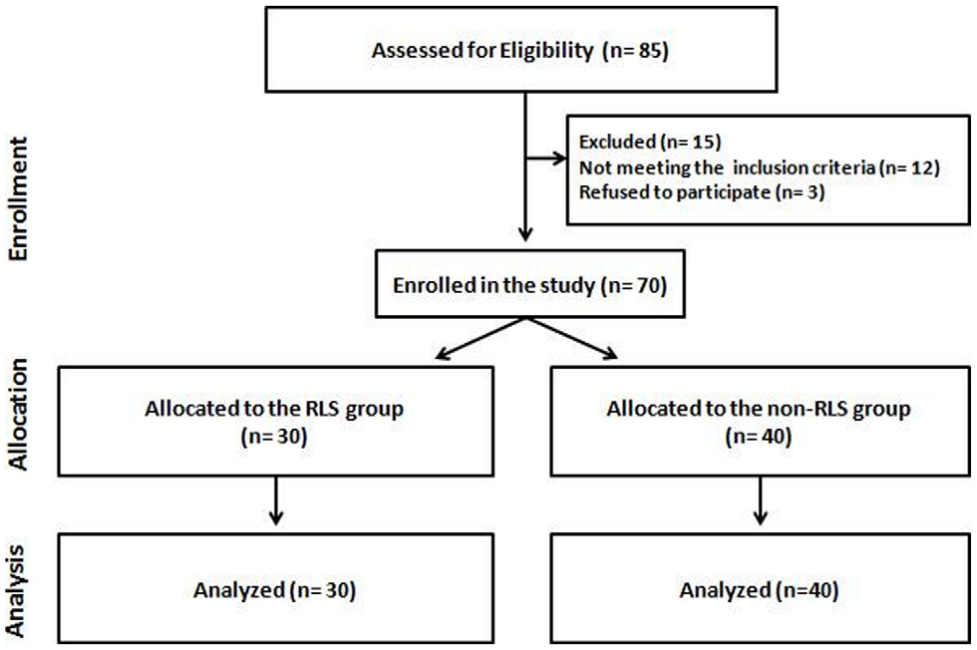
Patient recruitment flow diagram. Disposition of the patients into the two groups according to the RLS diagnosis as follows: the RLS group and the non-RLS group.

From September of 2006 to July of 2010, a total of eighty-five HD patients (the total number of patients receiving care in the study centers) were screened while seventy HD patients (51 male, 19 female, 54.1±16.9 years) were finally enrolled in the study (Figure 1). The patients were recruited from the HD units of the University Hospital of Larisa (UHL) and from the General Hospital of Trikala, Greece.

The inclusion criteria for the study were: dialysis for at least three months or more with adequate dialysis delivery and with stable clinical condition. In addition, none of the recruited patients should have been diagnosed with RLS prior to the study. Exclusion criteria included diagnosed neuropathies (n = 8, clinically examined by a neurologist) or reasons for being in a catabolic state (n = 4, including malignancies, opportunistic infections or active inflammation), within 3 months prior to the start of the study, or with abnormal C-Reactive Protein blood levels. In addition, another 3 patients refused to participate to the study for personal reasons.

### Allocation

Patients were divided into two groups according to their RLS status: the RLS group (n = 30; 10 females, 55.4±13.4 years) and the non-RLS group (n = 40; 9 females, 53.1±19.3 years).

### RLS diagnosis and severity assessment

RLS was diagnosed by a single RLS specialist neurologist, using the internationally recognised criteria of the IRLSSG [1], whereas the presence of Periodic Limb Movements in Sleep (PLMS) was derived from an overnight polysomnographic (PSG) study (Somnoscreen, Somnomedics GmbH, Randersacker, Germany). According the International RLS Study Group, the presence of PLMS is considered as a supportive and not conclusive clinical feature for the diagnosis of the syndrome and therefore it was used complementary to the IRLSSG criteria [1]. The assessment of PLMS was performed according to the official World Association of Sleep Medicine standards for recording and scoring of PLMS with a cut off threshold at >15 events per hour [18]. RLS patients were included in the study if they had at least one RLS episode per week excluding the episodes that took place during the dialysis session. Finally, RLS severity was assessed using the IRLSSG severity rating scale [19].

### Study design

This is a cross sectional study. Patients screening for RLS took place prior to any measurements and the status of the patients was coded for blinding purposes. All investigators were blinded to the RLS status of the patients up to the completion of data analysis. Patients were studied in a single out-patients visit on a free dialysis day.

### Body and muscle composition

A DEXA system (Lunar model DPX Madison, WI) was used to measure the patient's whole body fat and lean body mass [20]. *Post-hoc* regional analysis of the DEXA images was performed as described previously [21]. In addition, muscle size and composition of the right thigh was assessed by computed tomography (CT) (Philips Tomoscan SR5000) [22] with image analysis performed as previously described [16]. Waist to hip ratio (WHR) was calculated as waist circumference, at the midway between the iliac crest and the lowermost margins of the ribs, over the hip circumference at the maximum circumference of buttocks.

### Physical performance assessment

Patients' physical performance was evaluated by a battery of tests. Briefly, we used the “North Staffordshire Royal Infirmary” walking test (NSRI), two gait speed tests (normal and fast walk tests) and two sit-to-stand tests (STS-5 and STS-60) as previously described [23]–[25].

### Questionnaires

All questionnaires were completed with the interview method, by experienced personnel. The patient's subjective QoL outcomes were evaluated by using a Short Form-36 Health Survey (SF-36) version modified for patients receiving HD therapy [26]. Outcome scores can range from 0 to 100, with higher scores indicate better health status. The 8 multi-item scales of the SF-36 were summarized into two dimensions (mental and physical dimension respectively) and into a “total or overall SF-36 score” [26]. The patient's score in the physical health component was considered as the “physical functioning” score [27], [28].

The patients' depression levels were evaluated by using a 20-item self-rating depression scale questionnaire developed by Zung [29], used successfully in the past in HD patients [16], [30]. The cut-off point for the diagnosis of clinical depression is above 50.

The Epworth sleepiness scale (ESS) was used to assess the daily sleepiness level of the patients [31]. ESS consists of questions referring to eight situations. The patients are asked to rate on a scale of 0 up to 3 how likely they would be to doze off or fall asleep during the 8 situations. Outcome scores can range from 0 to 24. A score above 10 is considered to be pathological sleepiness.

A weekly sleep diary, adapted from the University of Massachusetts Medical School website (http://healthnet.umassmed.edu/mhealth/WeeklySleepQuestionnaire.pdf), was used to evaluate the patient's quality of sleep. Briefly, the sleep diary contained questions regarding how often during the previous week HD patients experienced any of the following: (1) difficulties falling asleep, (2) number of nocturnal awakenings, (3) difficulties remaining asleep, (4) the sensation of waking-up tired and fatigued, (5) day time stress and (6) how often did they feel refreshed after the night's sleep. The sleep diary was scored as follows: ‘never’ (0 points), ‘1–2 times a week’ (1 point), ‘3–5 times a week’ (2 points), ‘6–7 times a week’ (3 points). For question number 6 the scoring was reversed with 3 points for the answer ‘never’, and 0 points for the answer ‘6–7 times a week’. The sleep diary score was calculated as the sum of the total points with the minimum at zero points and the maximum score at 18.]

### Nutritional assessment

The nutritional status of the patients was examined by the Subjective Global Assessment (SGA) method [32].

### Biochemical assessment

Routine monthly laboratory results were recorded for HD subjects including ferritin, hematocrit, hemoglobin and dialysis efficiency parameters. A single-pool Kt/V was calculated from pre- and post-dialysis BUN measurements using the Daugirdas II equation [33]. The biochemical analysis was performed at the clinical lab of the UHL under standard hospital procedures.

### Hemodialysis procedure

Patients underwent HD therapy (Fresenius 4008B, Oberursel, Germany) for at least 4 hours, 3 times/week, with hollow-fiber dialysers and bicarbonate buffer. Low molecular weight heparin (Enoxaparin, Clexane®, Sanofi-Aventis, Strasbourg, France) was applied for anticoagulation. Enoxaparin doses of 40–60 mg were administered intravenously before the beginning of HD treatment. EPO therapy was given after the completion of HD session in order to normalize hemoglobin within 11-12 (g/dL).

### Statistical analysis

For the patients' characteristics (Table 1) an unpaired *t-*test were used to compare groups for continuous normally distributed variables; chi-square, for categorical variables; and Mann-Whitney *U* test, for non–normally distributed variables. Multivariate Analysis of covariance (MANCOVA) with “gender” as a covariance was used in order to control for possible differences between the examined variables (Tables 2–3). Spearman rank correlation test was used to assess the relationships between the examined variables, while for correlation between continuous and categorical variables a “Point Biserial Correlation Coefficient” test was used to assess this type of relationship. All analyses were carried out using the SPSS Statistical Package (SPSS 15.0, Chicago, Illinois). Data are presented as mean ± SD unless otherwise stated and the level for statistical significance was set at P<0.05.

**Table 1:** Patient's characteristics presented as pool data and divided in two groups according to RLS diagnosis.

Variables	Patients Pool Data	Non RLS	RLS	P values
N	70	40	30	-
Female / Male	19/51	9/31	10/20	0.313¥
Age (yr)	54.1±16.9	53.1±19.3	55.4±13.4	0.580
BMI (Kg/m^2^)	25.5±4.2	25.2±4.5	25.8±3.9	0.580
Kt/V	1.24±0.4	1.25±0.5	1.22±0.4	0.802
Years in Hemodialysis	3.0±2.6	2.5±1.6	3.7±3.5	0.093
WHR	0.97±0.07	0.97±0.06	0.97±0.07	0.744
PLMS prevalence	26 (37%)	9 (22%)	17 (57%)	**0.008** ¥
PLMS index (per hour)	20.4±29.3	10.3±20.2	31.5±33.7	**0.003**
IRLS score	-	-	23.7 ± 9.2	-
SGA (A/B/C)	31/9/1	22/7/1	9/2/0	0.820¥
Ferritin (ng/ml)	202.3±189.5	173.2±173.3	241.1±206.6	0.187
Hct	36.3±4.9	35.7±5.5	37.0±3.9	0.300
Hb (g/dL)	11.8±1.6	11.5±1.6	12.2±1.5	0.107

All data are mean ± SD. Abbreviations: BMI, body mass index; Kt/V, dialysis efficiency; WHR, waist to hip ratio; PLMS, Periodic Limb Movements in Sleep; IRLS, International Restless Legs Syndrome severity scale; SGA, subjective global assessment; Hct, hematocrit; Hb, hemoglobin.

¥ For categorical data a chi-square test was performed.

**Table 2:** Physical performance, depression, daytime sleepiness and sleep quality data.

Variables	Patients Pool Data	Non RLS	RLS	P values
**Physical Performance Tests**				
STS- 5 (sec)	10.1±2.8	10.4±3.3	9.7±1.9	0.330
STS-60 (rep)	27.8±8.4	28.8±8.9	26.6±7.6	0.339
Normal Walk (sec)	5.9±1.4	5.9±1.5	5.8±1.2	0.572
Fast Walk (sec)	4.1±1.0	4.1±1.1	4.1±0.9	0.687
NSRI test (sec)	84.3±36.8	85.6±41.4	82.7±30.5	0.503
**Questionnaires**				
Sleep Diary	7.2±4.8	5.7±4.3	9.2±4.7	0.005
Epworth Sleepiness Scale	6.1±4.1	5.3±3.7	7.1±4.4	0.060
Zung Depression Scale	41.0±9.6	38.2±8.3	44.4±10.1	0.011

All data are mean ± SD. P values are adjusted for yrs in dialysis. Abbreviations: STS-5, sit-to- stand test 5-repetitions; STS-60, sit-to- stand test 60 seconds; NSRI, North Staffordshire royal infirmary test.

**Table 3:** Body composition data.

Variables	Patients Pool Data	Non RLS	RLS	P values
**DEXA Body Composition Analysis**				
Total Body Fat (%)	27.4±11.3	25.3±11.9	29.7±10.5	0.554
Trunk Fat (%)	27.9±11.3	26.1±12.3	29.9±9.7	0.559
% Legs Fat	27.4±11.2	25.0±12.2	30.0±11.9	0.515
% Arms Fat	24.4±12.6	22.3±12.7	26.6±12.3	0.744
Total LBM (Kg)	46.1±8.8	47.3±9.2	44.9±8.3	0.985
Legs LBM (kg)	14.5±3.1	15.2±3.4	13.8±3.7	0.333
Arms LBM (kg)	5.0±1.4	5.5±1.6	4.6±1.0	0.091
**CT Thigh Analysis**				
Thigh Total Area (cm^2^)	112.1±25.5	119.4±28.9	104.3±18.8	**0.050**
EMCL CSA (cm^2^)	17.4±8.7	20.1±8.6	14.5±8.0	**0.022**
EMCL CSA/Total Body Fat	1.1±1.2	1.6±1.7	0.7±0.4	**0.048**
EMCL (%)	15.7±7.4	17.2±6.8	14.1±7.7	0.089
Muscle CSA (cm^2^)	94.5±25.2	101.4±28.8	87.1±18.4	0.066
Muscle CSA/Total LBM	2.1±0.4	2.2±0.4	1.9±0.3	**0.009**
Muscle (%)	82.7±6.9	82.4±6.8	83.1±7.2	0.187
Muscle (%)/EMCL (%)	7.4±5.3	6.3±3.1	8.4±6.7	0.145
SAT CSA (cm^2^)	115.0±54.1	113.5±56.7	116.5±52.5	0.723

All data are mean ± SD. P values are adjusted for yrs in dialysis. Abbreviations: LBM, lean body mass; EMCL, extramyocellular lipids (fat infiltration); CSA, cross sectional area; SAT, subcutaneous adipose tissue.

### Sample Size

Sample size calculations were conducted based in functional capacity [NSRI 73.5(30) vs 105(37)] and quality of life score [78.1(12.9) vs 63.7(20.2)] values in the hemodialysis patients from previous published articles [16], [34]. The resulting minimum required sample size was an average of 30 for 2-sided type 1 and type 2 errors of 5%.

## Results

In this cross sectional analysis, the prevalence of RLS within the HD population was approximately 42% (30 out of 70), whereas 57% of those (17 out of 30) co-experienced PLMS. The RLS patient's score in the IRLS severity scale was 24±9, which categorized them at the “severe” level of symptoms' intensity.

The prevalence of RLS was found to be higher in women compared to men (52% vs 39%), however no gender effect was observed in the current analysis (P = 0.313).

**Figure 2:**
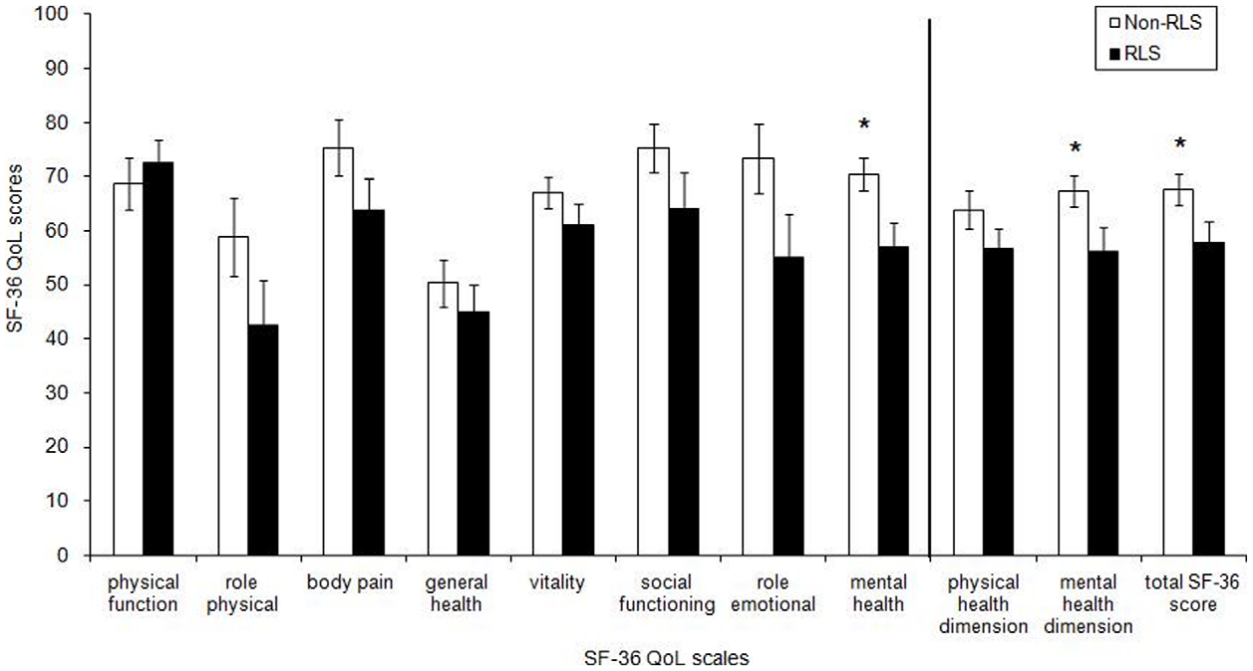
Health-related Quality of Life scores for the RLS (in black) and non-RLS (white bars) hemodialysis patients. Vertical line: The first two sets of bars on the right of the vertical bar are the summary physical and mental dimension score while the last set of bars at the far right is the total SF-36 score.

The patients' characteristics are presented in Table 1 and physical performance data are presented in Table 2. There were no significant differences between the two groups in all direct physical performance tests performed. In contrast, significant differences were found in the QoL assessment results (Figure 2), in the sleep diary [F (1,60) = 8.472, P = 0.005], in the severity of the Zung self-rating depression scale [F (1,60) = 6,922, P = 0.011] and a trend in the sleepiness scale [F (1,60) = 3,656, P = 0.060] (Table 2).

The severity of RLS symptoms ‘score (IRLS) was correlated significantly with the score in sleep diary (r = 0.410, P = 0.030). The prevalence of RLS was correlated with sleep diary (r_pb_ = +0.31, P = 0.01), and Zung depression scale scores (r_pb_ = +0.29, P = 0.01), whereas significant negative correlations were found between the IRLS score and the overall score of the SF-36 (r = −.0423, P = 0.022).

Total body composition assessment did not show any differences between the two groups. The CT analysis showed that the thigh muscle total area, muscle cross sectional area (CSA) and the level of muscle fat infiltration (EMCL-extramyocellular lipids) were significantly reduced in the RLS group (Table 3). In addition, muscle CSA/total LBM ratio and EMCL CSA/total body fat ratio were found to be significantly increased in the non-RLS group [F (1,41) = 7.538, P = 0.009] and [F (1,41) = 4.130, P = 0.048] respectively, whereas, no significant differences were found in the percentage of muscle and EMCL between the two groups [F(1,41) = 2.207, P = 0.145].

## Discussion

A 42 % of the examined patients were diagnosed with RLS in agreement with other recent studies [35]. To the best of our knowledge this is the first study assessing parameters that affect survival and quality of life in such detail in patients with uremic RLS. We found that the size of proximal muscles was significantly reduced in the RLS group, however, uremic RLS did not seem to have an additional detrimental impact on the already diminished physical performance, body and muscle composition and physical aspects of the quality of life in HD patients. The low level of QoL reported by the RLS- HD patients seems to be due mainly to mental health and sleep related aspects rather than the physical aspects.

It is well known that HD patients are characterized by low VO_2_ peak,[9] reduced physical performance, high levels of weakness and exercise intolerance [36]. In the RLS population with normal kidney function, maximal oxygen uptake values [37] and physical fitness levels [38] have been found to be lower compared to non-RLS healthy individuals. It is, therefore, plausible to hypothesize that HD patients with RLS could have been affected in an additive or synergistic way by uremia and RLS, and that this combination would affect their functional capacity and physical performance. In the current study patients of both groups responded similarly in all physical performance tests, implying that there was no discernible effect of RLS on physical performance in patients receiving dialysis therapy. In agreement to our findings, a study that assessed elderly idiopathic RLS patients using the same methodology with us, found no significant differences in physical performance between sufferers and non-sufferers [39].

Moreover, it is known that HD patients are characterized by significant reductions in muscle size and increased muscle fat infiltration [40]. The analysis of CT images revealed among other that the total thigh area was reduced by 15%, the muscle CSA by 17% and the EMCL CSA area by 38% in the RLS-HD patients. This reduction of muscle mass is worrisome especially since muscle atrophy is usually prominent in HD patients and has been related to high mortality [11]. Independently from the increased levels of atrophy however, there were no visible changes in the muscle composition between groups and this could be speculated to be a positive counteracting effect induced by the increased muscle activity in which RLS patients involuntarily engage. The reduction of total thigh muscle area in the RLS group could be partially explained by the respective reductions in both EMCL and muscle CSAs that were found in that group and not by the amount of subcutaneous fat tissue as this appeared to be similar in both groups.

The differences in the EMCL area in the RLS patients could be a result of an increased fat oxidation due to higher muscle activity, as it is observed in highly trained athletes [41] however this is only a hypothesis and it should be seen with caution. The fact that no-significant differences were observed in the nutritional status within the two groups, could indicate that the observed differences in the HD-RLS patient's muscle size were not caused by malnutrition. Overall, it seems that the observed changes in muscle size do not have the magnitude to worsen functionality and further reduce physical activity in HD patients with RLS.

RLS has been blamed also for sleep deprivation [13] which in turn could evoke alterations in anabolic hormones secretion and circulation such as in growth hormone (GH) and insulin-like growth factor I (IGF-I) [14], [15] eventually affecting the patient's anabolism and muscle mass. Subjective sleep quality was found reduced by 61% (sleep diary) reinforcing the hypothesis that the lack of restorative sleep could be one of the factors affecting muscle mass. The RLS-induced sleep deprivation is an important health issue that needs to be addressed further in order to reduce all the possible factors contributing to muscle atrophy and accelerated catabolism in HD patients.

Depression symptoms are common in patients receiving HD therapy [42], [43]. It is plausible that HD patients with RLS might experience higher levels of depression as a result of their restless night, the related insomnia and the overall reduced QoL that RLS is associated with [44]. Indeed, the RLS group showed higher depression score than the non-RLS group confirming previous data [45], [46], and the RLS status significantly correlated with the score in the Zung depression scale. Even thought depression score was elevated in both groups, none of them exceeded the threshold for the diagnosis of clinical depression (cut off<50). It is also known that low sleep quality exacerbates the severity of depression symptoms seen in HD patients [47] and this is in agreement with our findings. Given that the sleep quality in the HD patients with RLS is significantly impaired compared to the HD patients without the syndrome [5], this could explain in part the differences in depression symptoms severity score between the two groups. This also agrees with our PLMS results, as PLMS were two fold increased in the RLS HD patients affecting even more their sleeping pattern.

So far it was not clear whether the impaired QoL seen in RLS HD patients was due to physical or mental QoL components. A closer look at the questionnaires as well as the physical performance tests reveals that the differences between the two groups are better explained by their diminished mental rather than their physical aspects, confirming data derived from previous studies in RLS in HD patients [5], [6]. To the best of our knowledge ours is the first study to carefully investigate, by using both indirect (surveys-questionnaires) and direct (physical performance tests) approaches, what quality of life aspects are the most affected in these patients. The revealed consequences seem to be common also for other symptomatic forms of the sleep disorder (e.g. RLS due to type 2 diabetes). In the past, Merlino et al. concluded that patients accustomed to severe physical dysfunctions (eg, uremia and diabetes) complain of RLS more as a mental distress than a physical one [48] without however providing measurable indices (e.g. functional tests) of the presence or lack of physical impairments. The current study reports a higher level of depression and even poorer sleep quality in the HD patients with RLS compared to their RLS-free counterparts.

RLS severity has been associated with lower survival [8] while PLMS severity is considered an independent predictor of mortality [49]. In the current study, the intensity of the syndrome in our RLS patients was categorized as “severe” [19] implying that those patients might be subjects to a higher CVD risk. However, such a claim will have to be further validated, as it was not straightforward substantiated by our findings.

### Limitations

It has been reported that PLMS index can vary across nights in patients with idiopathic RLS [50] but it is not known if the same phenomenon exists in patients with secondary type of RLS. Thus, in that respect our data should be viewed with caution since the PLMS indices obtained by a single overnight PSG study may be not as reliable as if we had used an average of two or even three consecutive assessment nights.

In conclusion, hemodialysis patients with RLS reported lower quality of life and overall scored worse in mental rather than physical parameters compared to their RLS free counterparts. The size of proximal muscles was found to be significantly reduced in the RLS group, however, uremic RLS did not seem to have an additional detrimental impact on the already diminished physical performance, body and muscle composition and physical aspects of the quality of life in HD patients. It is possible that the potential negative impact of RLS on the above parameters could have been masked under the dominant impact of uremia and/or hemodialysis *per se*. Screening for RLS should be part of the routine general health assessment of the hemodialysis patients as it affects QoL and possibly overall health status.
